# Case Report: Unclassifiable cerebellar high-grade neuroepithelial tumor with a *CCDC6*::*RET* fusion manifesting explosive recurrence

**DOI:** 10.3389/fsurg.2026.1709333

**Published:** 2026-04-08

**Authors:** Moksada Regmi, Shikun Liu, Ying Xiong, Junyi Liu, Zihan Zhao, Xu Zhang, Chenlong Yang, Sino-Uzbek Regenerative Therapy Consortium (SURE)

**Affiliations:** 1 State Key Laboratory of Vascular Homeostasis and Remodeling, Department of Neurosurgery, Peking University Third Hospital, Peking University, Beijing, China; 2 Center for Precision Neurosurgery and Oncology of Peking University Health Science Center, Peking University, Beijing, China; 3 Peking University Health Science Center, Beijing, China; 4 Center for Oculocranial Pressure Instability Disorders, Henan Academy of Innovations in Medical Science (AIMS), Zhengzhou, China; 5 Shenzhen Graduate School, Peking University, Shenzhen, China

**Keywords:** high-grade, Ki-67, leptomeningeal dissemination, neuro-oncology, tumor

## Abstract

A 20-year-old woman presented with a 1-month history of positional vertigo, occipital headaches, and progressive gait ataxia. Neuroimaging demonstrated a 3.7 × 4.2 × 3.3 cm heterogeneously enhancing mass in the left cerebellar hemisphere with fourth-ventricle compression, approximately 8 mm tonsillar herniation, and obstructive hydrocephalus. Urgent resection was performed through a midline suboccipital approach with neuronavigation and fluorescein guidance, achieving gross total removal. Histopathology showed an undifferentiated high-grade neuroepithelial malignancy with brisk mitotic activity and necrosis. The Ki-67 labeling index exceeded 80% in hotspot regions. Immunophenotyping showed partial glial marker expression and neuroendocrine marker expression; INI1 was retained and other markers did not support more common defined entities. Hybrid-capture DNA and RNA next-generation sequencing identified *TP53* c.524G > A (p.R175H), amplification of *CDK4* and *AURKA*, and an in-frame *CCDC6* (exon 1)::*RET* (exon 12) fusion. Based on the integrated histologic, immunophenotypic, and molecular findings available, the tumor could not be assigned to a specific WHO-defined CNS tumor entity. Despite gross total resection and postoperative oncologic management, the tumor recurred rapidly with leptomeningeal dissemination and progressed to multifocal posterior fossa and brainstem disease. The patient died five months after diagnosis. This case illustrates the diagnostic and therapeutic challenges posed by rare, highly aggressive CNS neoplasms that do not map to a WHO-defined entity on available testing and highlights the potential clinical relevance of identifying actionable *RET* fusions in high-grade neuroepithelial tumors.

## 1 Background

High-grade cerebellar tumors in young adults are uncommon and represent a small fraction of CNS malignancies (1). Proliferation indices such as Ki-67 correlate with grade and outcome in multiple CNS tumor types; reported ranges for glioblastoma and medulloblastoma vary widely across series and methods, but hotspot labeling approaching or exceeding 80% is unusual and typically aligns with highly aggressive clinical behavior (2). At the same time, Ki-67 interpretation is constrained by intratumoral heterogeneity, hotspot bias, and interobserver and technical variability, which can reduce incremental prognostic discrimination once a tumor is clearly high grade.

The WHO CNS classification relies on an integrated diagnosis that combines histology with defined molecular alterations to assign entities and, where applicable, grades. A subset of cases still cannot be placed into a specific WHO-defined entity when key defining alterations are absent, conflicting, technically unavailable, or biologically ambiguous, and such cases may pose challenges for prognostication and treatment selection.

Actionable kinase fusions are a well-established therapeutic paradigm in several systemic cancers. *RET* fusions in lung and thyroid cancers can be targeted with selective inhibitors such as selpercatinib, including reported intracranial activity in metastatic settings (3). In primary CNS tumors, *RET* fusions are rare, and the clinical value of *RET* inhibition in this context remains uncertain (4).

Here, a high-grade posterior fossa neuroepithelial tumor in a young adult showed very high proliferative activity and an activating *CCDC6::RET* fusion on DNA and RNA sequencing, while the integrated pathologic and molecular profile did not satisfy criteria for a specific WHO-defined entity. The clinical message is that in diagnostically ambiguous posterior fossa tumors with explosive clinical progression, early DNA and RNA profiling may reveal targetable alterations that influence trial eligibility and targeted therapy considerations.

## 2 Case presentation

A 20-year-old woman with no significant medical history presented with 5 weeks of progressive vertigo and occipital headaches. The vertigo was positional, worse when lying flat, and relieved on sitting upright. She denied vision loss, syncope, or limb numbness. The occipital headaches were dull and throbbing and only minimally responsive to analgesics. Two weeks before admission, her symptoms worsened with near-constant dizziness, daily nausea, intermittent vomiting, and new gait unsteadiness. By the time of presentation, she required assistance to stand and walk.

On examination, the patient was alert and oriented. Cranial nerve testing was normal, and there was no papilledema on fundoscopic examination. Motor and sensory examinations of the limbs were unremarkable. She had pronounced truncal ataxia, difficulty sitting unsupported, and impaired limb coordination bilaterally on finger-to-nose and heel-to-shin testing. Vital signs were within normal limits. There were no stigmata of neurocutaneous syndromes. Her family history was noncontributory, and she had no known toxin or radiation exposures.

**Figure 1:**
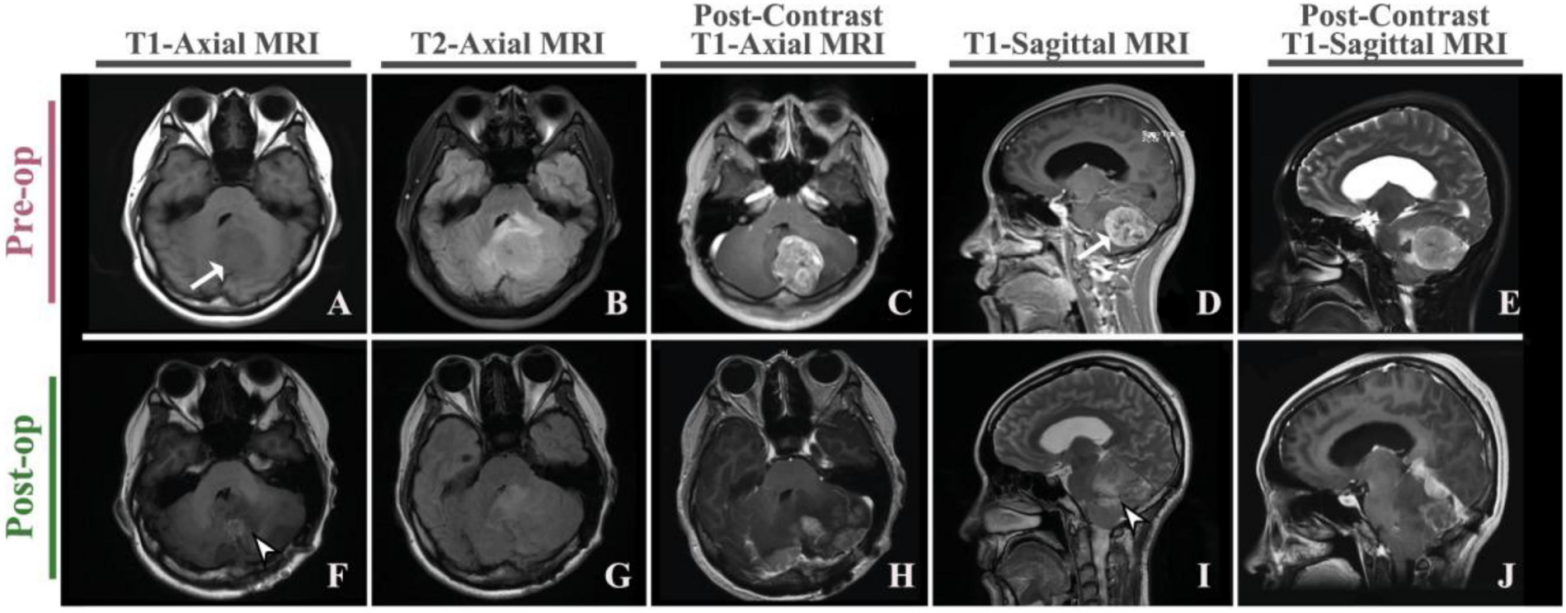
Pre- and postoperative radiological findings. (**A**) T1-weighted axial (preoperative); (**B**) T2-weighted axial (preoperative); (**C**) post-contrast T1-weighted axial (preoperative); (**D**) T1-weighted sagittal (preoperative); (**E**) post-contrast T1-weighted sagittal (preoperative). Arrows in preoperative panels indicate the left cerebellar hemispheric mass. (**F**–**J**) Corresponding postoperative sequences at matched planes demonstrating the resection cavity without definite residual enhancement; arrowheads indicate the postoperative cavity/resection bed (slice positions matched).

An urgent brain MRI obtained at an outside hospital two days before transfer revealed a mass in the left cerebellar hemisphere with obstructive hydrocephalus. On arrival to our center, repeat high-resolution MRI with contrast showed a 3.7 × 4.2 × 3.3 cm mass in the left cerebellar hemisphere, heterogeneous on T1- and T2-weighted sequences with patchy enhancement (Figures 1A–E). Internal foci of cystic change and areas of susceptibility consistent with hemorrhage or calcification were present. There was extensive surrounding vasogenic edema in the cerebellar white matter. The mass severely effaced the fourth ventricle, displaced the brainstem, and resulted in approximately 8 mm of downward herniation of the cerebellar tonsils through the foramen magnum. The third and lateral ventricles were dilated, consistent with obstructive hydrocephalus. No supratentorial parenchymal lesions were seen aside from ventricular enlargement.

Lumbar puncture was deferred because of mass effect and herniation risk. Routine laboratory tests on admission showed mild hyponatremia (128 mmol/L) and hypokalemia (3.0 mmol/L), attributed to poor oral intake, and these were corrected. The complete blood count showed mild anemia (hemoglobin 9.1 g/dL). Serum tumor markers were unremarkable. Dexamethasone was initiated for radiologic and clinical evidence of raised intracranial pressure, with partial symptomatic improvement.

Because neuroendocrine marker expression broadened the differential, contrast-enhanced CT of the chest, abdomen, and pelvis was performed and did not identify an extracranial primary lesion.

## 3 Treatment and intraoperative findings

In view of progressive symptoms, cerebellar tonsillar descent, and obstructive hydrocephalus, neurosurgical intervention was prioritized. After expedited preoperative assessment and informed consent, the patient underwent a suboccipital craniotomy on hospital day 5. A midline posterior fossa approach was used, with stereotactic neuronavigation based on preoperative MRI and CT and intraoperative fluorescein sodium to help delineate tumor margins.

On dural opening, the left cerebellar hemisphere appeared tense and swollen. The tumor was encountered within the cerebellar parenchyma and had a grayish-red, friable appearance with indistinct borders. It was highly vascular, with engorged arterial feeders and brisk bleeding, and contained areas of necrosis. The tumor invaded the overlying dura mater, a finding that broadened the intraoperative differential. Under the operating microscope, areas of the tumor showed bright fluorescence after fluorescein injection, assisting identification of involved tissue.

Gross total resection of all fluorescent and grossly abnormal tissue was achieved, and postoperative contrast-enhanced MRI demonstrated no definite residual enhancement (Figures 1F–J). Critical structures, including the brainstem, cranial nerves, and deep cerebellar nuclei, were preserved. The fourth ventricle was decompressed and cerebrospinal fluid pathways were reestablished. An external ventricular drain was placed for temporary cerebrospinal fluid diversion.

Postoperatively, the patient was monitored in the ICU. She awoke without new neurological deficits; her pre-existing ataxia persisted, but vertigo and headache improved substantially, consistent with relief of hydrocephalus. The external ventricular drain was weaned and removed on postoperative day 3 without recurrence of hydrocephalus. There were no wound complications. She was discharged home four weeks postoperatively with plans for postoperative oncologic evaluation and treatment. After discharge, her condition deteriorated rapidly with radiographic progression and leptomeningeal dissemination, and she died five months after diagnosis.

## 4 Investigations and pathology findings

### 4.1 Histopathology

**Figure 2:**
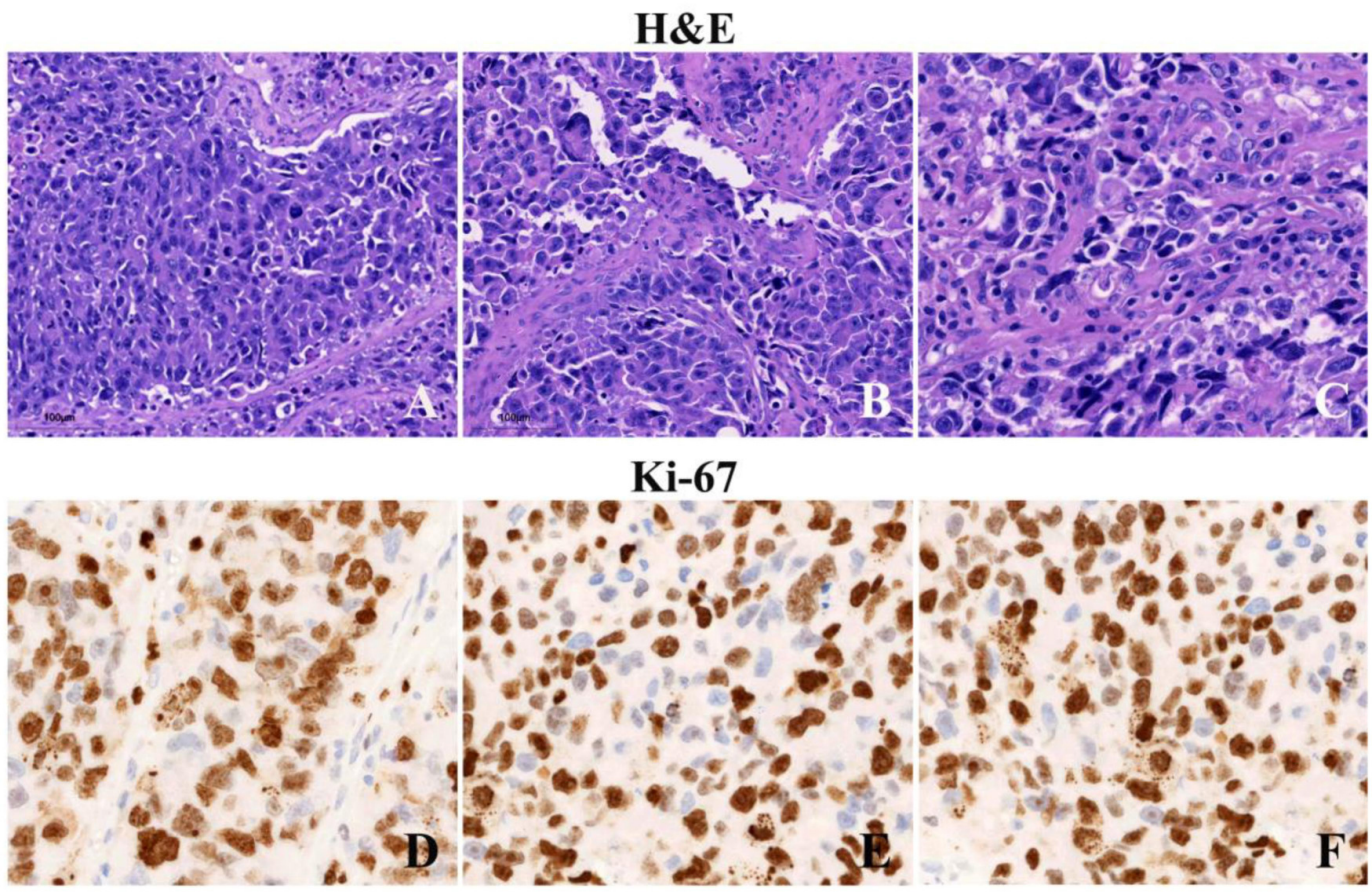
Pathological findings. Hematoxylin–eosin sections highlight undifferentiated epithelioid cytology with marked atypia, brisk mitoses, and tumor necrosis in (**A**–**C**). Hotspot fields demonstrating very high Ki-67 proliferation with dense nuclear immunoreactivity; the distribution is diffuse with only rare negative tumor nuclei in (**D**–**F**).

Formalin-fixed, paraffin-embedded sections showed an undifferentiated high-grade neuroepithelial malignancy composed predominantly of epithelioid cells with high nuclear-to-cytoplasmic ratios, marked nuclear pleomorphism, and brisk mitotic activity (Figures 2A–C). Tumor necrosis was present with palisading of tumor cells at the margins of necrotic foci. Definite rosettes or pseudorosettes were not identified.

### 4.2 Immunohistochemistry (IHC)

**Figure 3:**
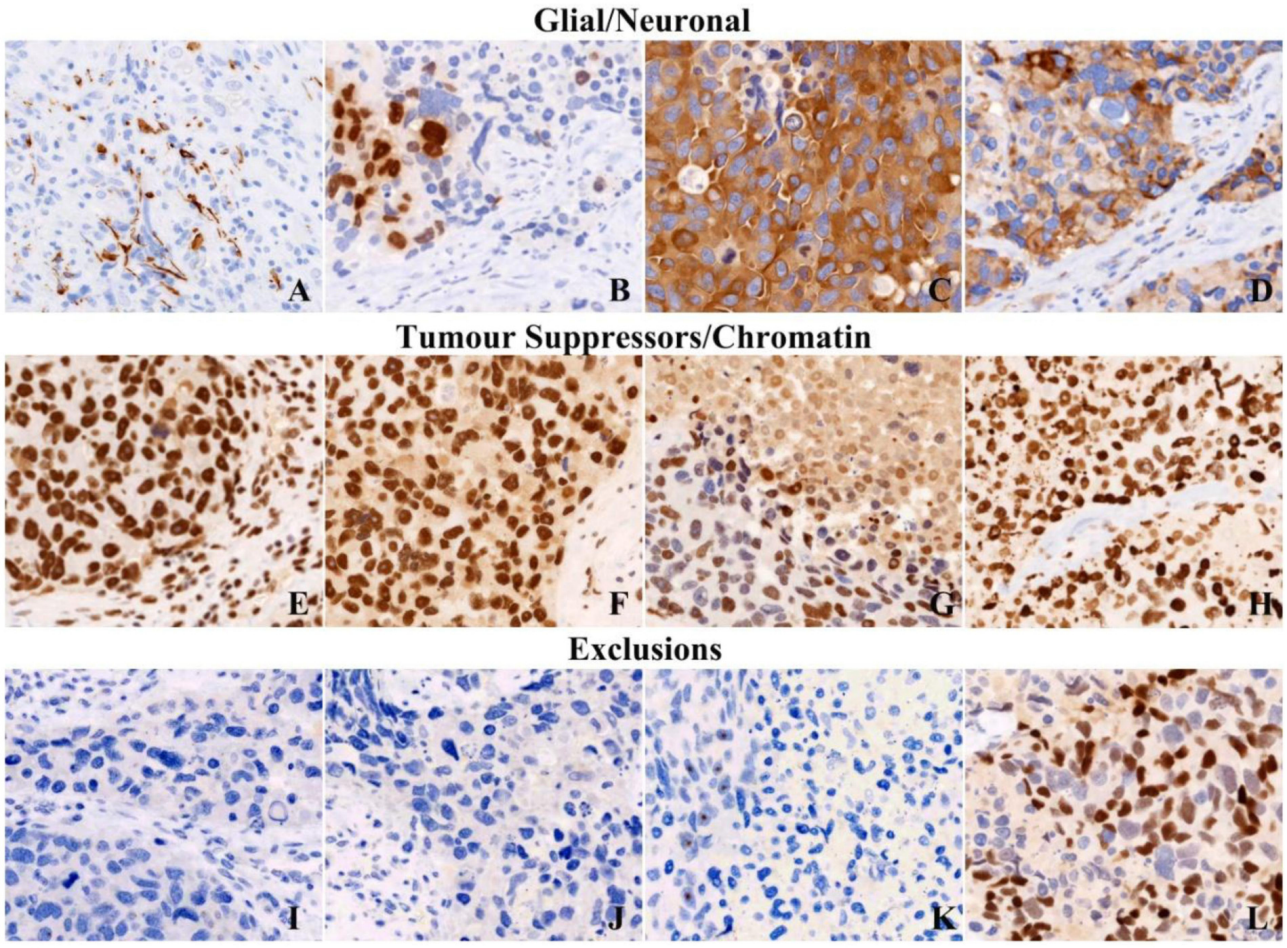
Immunohistochemical profile. Panels show scant GFAP positivity that may reflect reactive or entrapped astrocytes (**A**), Olig2 partial positivity (**B**), MAP2 positivity (**C**), synaptophysin diffuse weak positivity (**D**), INI1 retention (**E**), BRG1 retention (**F**), BRM partial loss (**G**), H3K27me3 retention (**H**), EMA negativity (**I**), pan-CK/CK-mix negativity (**J**), CD20 negativity supporting exclusion of lymphoma (**K**), and INSM1 partial positivity (**L**).

Immunohistochemistry demonstrated a mixed profile with partial glial marker expression and neuroendocrine marker expression. GFAP showed scant positivity that could reflect reactive or entrapped astrocytes, while Olig2 was positive in a subset of tumor cells (Figures 3A,B). MAP2 was positive (Figure 3C). Synaptophysin was weakly but diffusely positive (Figure 3D). The Ki-67 labeling index was markedly elevated, exceeding 80% in hotspot regions (Figures 2D–F). Considering intratumoral heterogeneity and the use of hotspot-based quantification, this finding supports an extremely high proliferative fraction and should not be regarded as a precise average across the entire tumor.

INI1 nuclear expression was retained (Figure 3E). BRG1 was retained (Figure 3F), while BRM showed patchy loss (Figure 3G). H3K27me3 was retained (Figure 3H), and H3 K27M mutant protein was negative. ATRX expression was retained and IDH1 R132H was negative by immunohistochemistry.

Markers that would support embryonal tumor entities were not expressed, including LIN28A, SALL4, and BCOR, and beta-catenin did not show nuclear accumulation. Lymphoid markers (CD3, CD20) were negative. Epithelial markers, including EMA and pan-cytokeratin (CK-mix), were negative (Figures 3I–K). INSM1 was positive in a subset of cells (Figure 3L), and CD56 and chromogranin A showed focal positivity.

The complete immunohistochemical panel is summarized in Table 1.

**Table 1:** Summary of immunohistochemical and molecular profiling.

Marker	Result	Comment
Ki-67	>80% (hotspot)	Ultra-high proliferation
GFAP	Focal +	Glial differentiation
Olig2	Partial +	Glial lineage
MAP2	+	Neuronal
Synaptophysin	Diffuse weak +	Neuroendocrine tendency
INSM1	Partial +	Neuroendocrine marker
INI1/BRG1	Retained	Rules out ATRT & *SMARCA4*-deficient
BRM (*SMARCA2*)	Partial loss	SWI/SNF dysfunction
H3K27me3	Retained	Argues against H3K27-altered entities
EMA/CKs	Negative	Non-epithelial
Lymphoid panel (CD20/CD3)	Negative	Lymphoma unlikely
Melanocytic (HMB45/Melan-A)	Negative	Melanocytic tumour unlikely
NGS	*TP53* mut; *CDK4*/*AURKA* amp; *CCDC6::RET* fusion	Actionability uncertain in CNS
Classifier	No confident match	WHO CNS5 NOS

### 4.3 Molecular and genetic testing

Given the unusual morphology and immunophenotype, hybrid-capture DNA and RNA next-generation sequencing was performed on FFPE tissue. The assay covered 571 genes at the DNA level and 2660 genes at the RNA level and assessed single-nucleotide variants and small insertions or deletions, copy-number alterations, and gene fusions.

Sequencing identified *TP53* c.524G > A (p.R175H). Copy-number analysis showed amplification of *CDK4* and *AURKA*. RNA sequencing identified an in-frame *CCDC6* (exon 1)::*RET* (exon 12) fusion. Additional molecular summary metrics, including tumor mutational burden and microsatellite instability assessment, are reported in Supplementary Table S1. The full gene list for the sequencing panel is provided in Supplementary Table S2.

**Figure 4:**
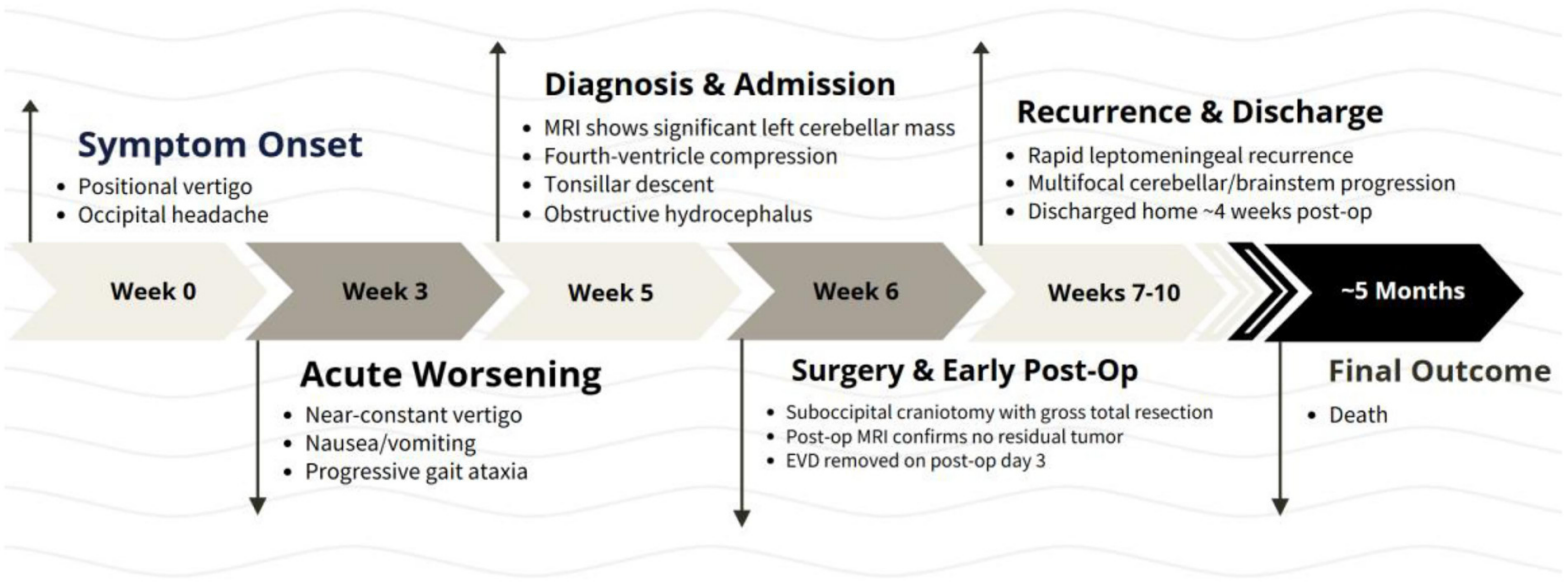
Clinical timeline of disease course. Chronological summary of symptom onset, diagnostic findings, surgical management, postoperative course, recurrence, and outcome.

DNA methylation-based classification can refine diagnosis in challenging cases (5); however, classifier output was not available for review in this case. After multidisciplinary review integrating histology, immunophenotype, and DNA and RNA sequencing results, the tumor was best classified as a high-grade neuroepithelial tumor, not otherwise specified, with neuroendocrine marker expression. A defined WHO entity (WHO CNS5) could not be assigned on the basis of available findings. The clinical timeline is summarized in Figure 4.

## 5 Discussion

This case describes an aggressive posterior fossa malignancy in a young adult that could not be assigned to a WHO CNS5-defined entity based on the available histologic, immunophenotypic, and molecular findings (6). Such cases create practical challenges in diagnosis, prognostication, and treatment selection, especially when clinical behavior is explosive.

The proliferative activity was striking, with Ki-67 labeling exceeding 80% in hotspot fields. High Ki-67 indices correlate with higher grade and shorter survival in gliomas and embryonal tumors (7). At the same time, Ki-67 assessment is limited by hotspot bias and intratumoral heterogeneity, and values above approximately 40% to 50% may provide limited additional prognostic discrimination within already high-grade tumors. In this context, the very high Ki-67 supports aggressive biology rather than providing a precise quantitative predictor of outcome. A second independent neuropathology review was not performed, which is acknowledged as a limitation.

Genomic profiling identified alterations with potential therapeutic relevance. The *CCDC6::RET* fusion is structurally consistent with an activating *RET* rearrangement, and *CCDC6* is a recognized *RET* fusion partner in other malignancies (4). In non-CNS cancers, selective *RET* inhibitors such as selpercatinib produce robust and durable responses, including intracranial responses in patients with brain metastases. This supports considering *RET* fusion status as clinically relevant in primary CNS tumors if adequate CNS exposure can be achieved, although direct evidence in this setting remains limited. Amplification of *CDK4* with co-amplification of *AURKA* implicates dysregulation of the *CDK4*/6–RB axis, a frequent event in high-grade gliomas (8). Phase II trials of *CDK4*/6 inhibition, including palbociclib, have shown limited benefit in recurrent glioblastoma (9), likely reflecting pharmacokinetic limitations and the need for rational combinations. Nonetheless, these alterations remain of interest as potential targets in the context of brain-penetrant *CDK4*/6 inhibitors and combination strategies.

The patchy loss of BRM (*SMARCA2*) suggests possible SWI/SNF complex dysfunction. Loss-of-function alterations in SWI/SNF components can create vulnerabilities to EZH2 inhibition, and EZH2 inhibitors such as tazemetostat are used in selected SWI/SNF-deficient tumors (10, 11). In this case, *SMARCA2* loss was patchy rather than complete and no truncating alteration was identified, so any implication of EZH2 inhibitor sensitivity is hypothesis-generating rather than actionable.

The biological significance of neuroendocrine marker expression warrants caution. Here, the pattern is most consistent with partial or aberrant marker expression within a high-grade CNS tumor rather than definitive evidence of an extracranial neuroendocrine lineage, particularly given negative systemic imaging. Even so, this immunophenotype can broaden the differential diagnosis and appropriately prompts systemic evaluation to exclude metastasis, as performed in this case.

Immunotherapy is also unlikely to be beneficial based on the available data. Low tumor mutational burden and no evidence of mismatch repair deficiency are features generally associated with limited response to immune checkpoint blockade. Large trials of anti-PD-1 or anti-PD-L1 therapy in unselected glioblastoma cohorts have not demonstrated survival benefit over standard therapy (12, 13).

Overall, this case supports early, comprehensive molecular workup in young adults with high-grade posterior fossa tumors, especially when histology and immunophenotype do not map to a defined entity. Early integration of targeted NGS and RNA sequencing can refine the differential diagnosis, identify potentially actionable alterations, and support enrollment in molecularly stratified clinical trials or compassionate-use pathways. Given the rarity and heterogeneity of such tumors, basket trials and n-of-1 approaches that allocate treatment by molecular features rather than histologic labels may be particularly relevant.

## 6 Conclusions

This report describes a young adult with a high-grade cerebellar neuroepithelial tumor showing very high proliferative activity, an in-frame *CCDC6* (exon 1)::*RET* (exon 12) fusion, and rapid leptomeningeal dissemination with short survival despite gross total resection. Comprehensive profiling was essential for excluding defined WHO entities and for identifying potentially targetable alterations, although agents directed against *RET* and *CDK4* are not yet of proven benefit in primary CNS disease. Larger multicenter series and prospective, molecularly stratified clinical trials are needed to clarify recurrence patterns, refine prognostic assessment, and test targeted strategies for this rare and challenging group of tumors.

## Data Availability

The original contributions presented in the study are included in the article/Supplementary Material, further inquiries can be directed to the corresponding author.
