# Supplementary figures and images for: Case Report: Unclassifiable cerebellar high-grade neuroepithelial tumor with a CCDC6::RET fusion manifesting explosive recurrence
Source: Front Surg. 2026 Apr 8;13:1709333. doi: 10.3389/fsurg.2026.1709333 (PMC13099530; doi:10.3389/fsurg.2026.1709333)

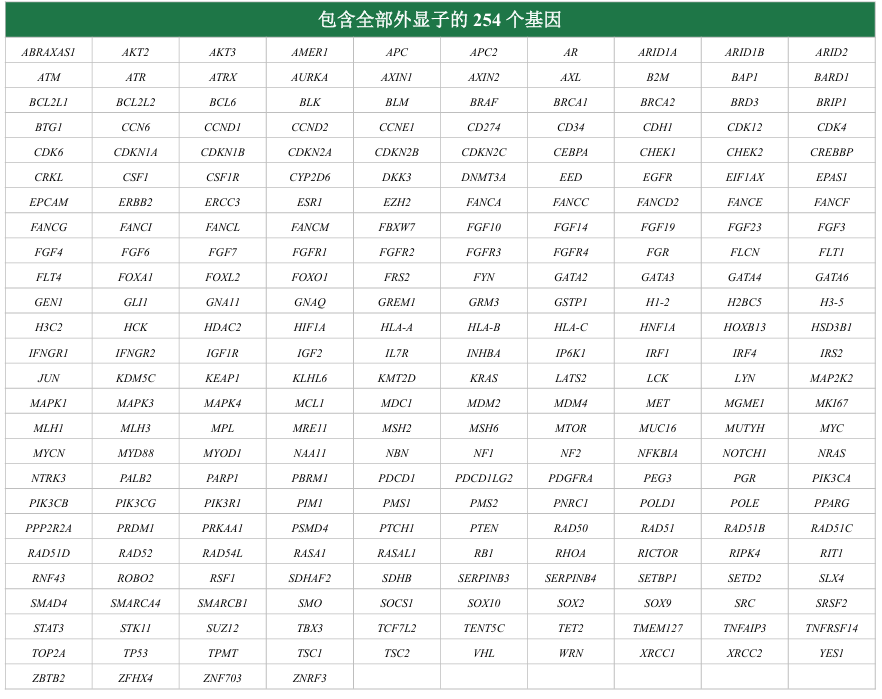

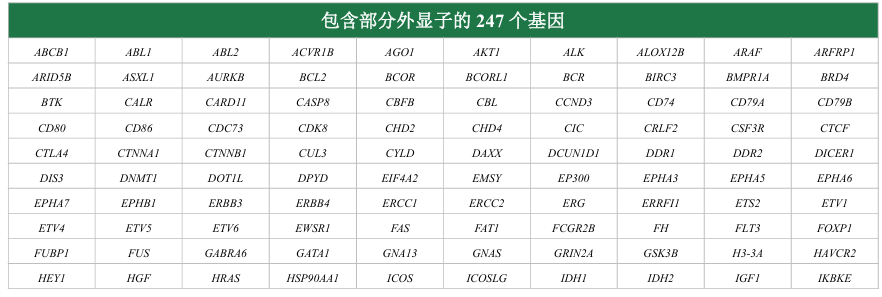

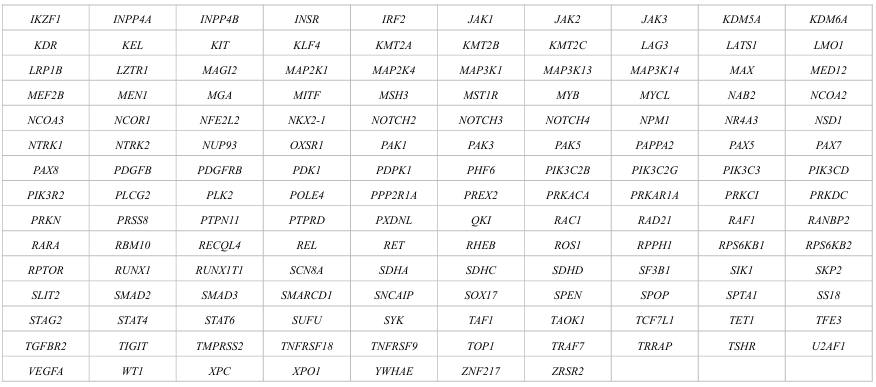

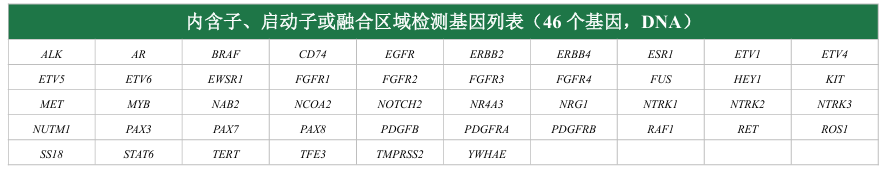

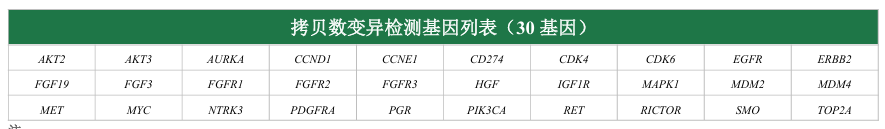

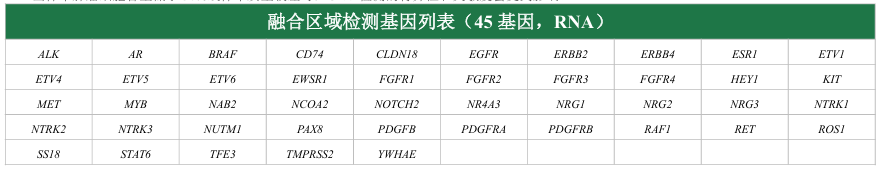

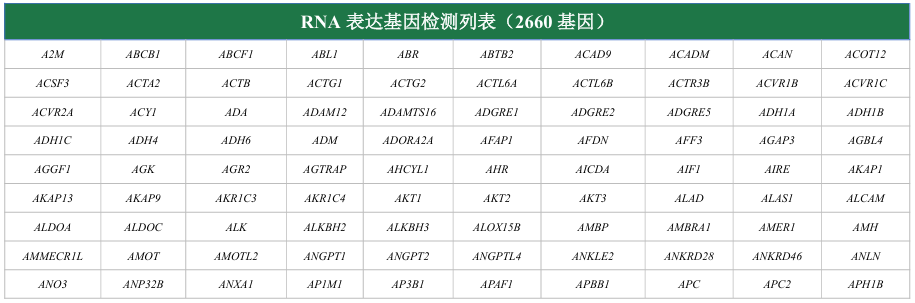

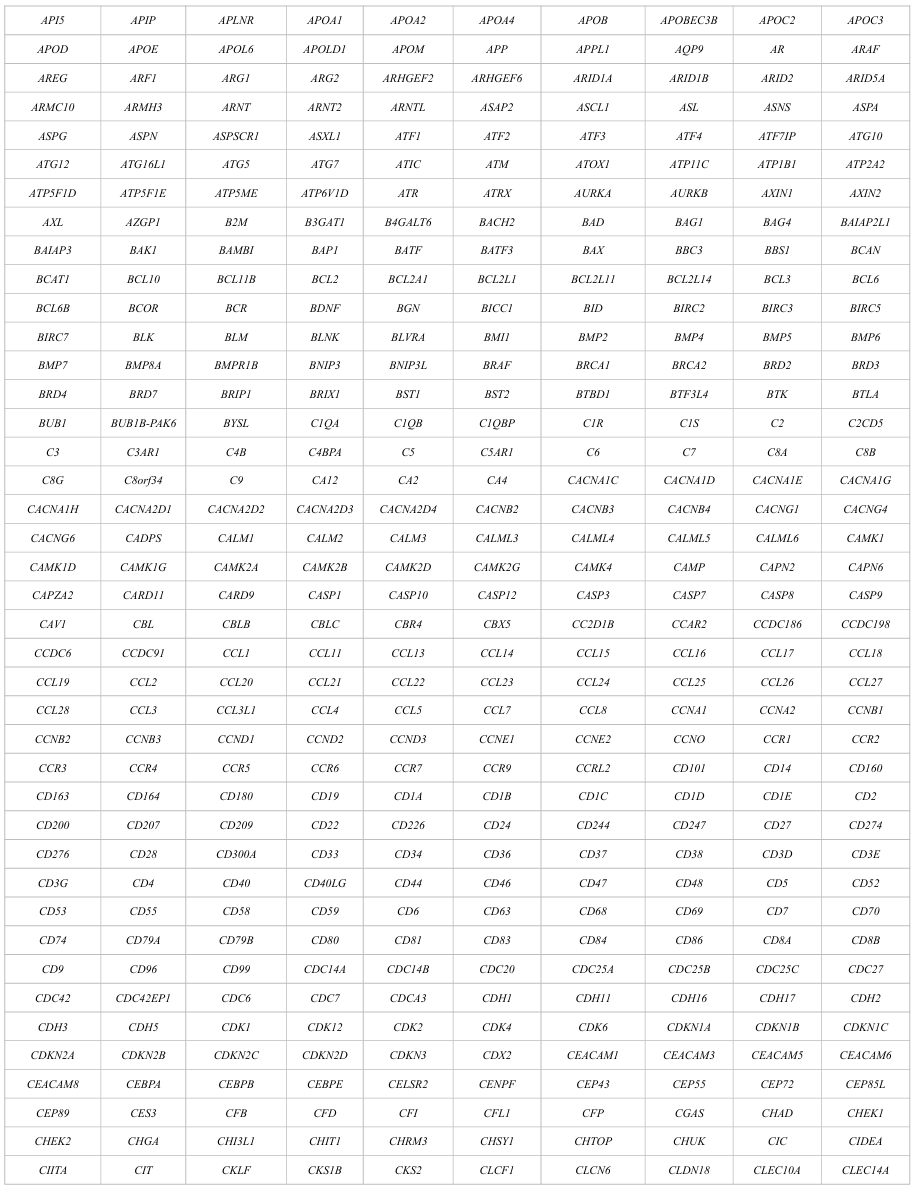

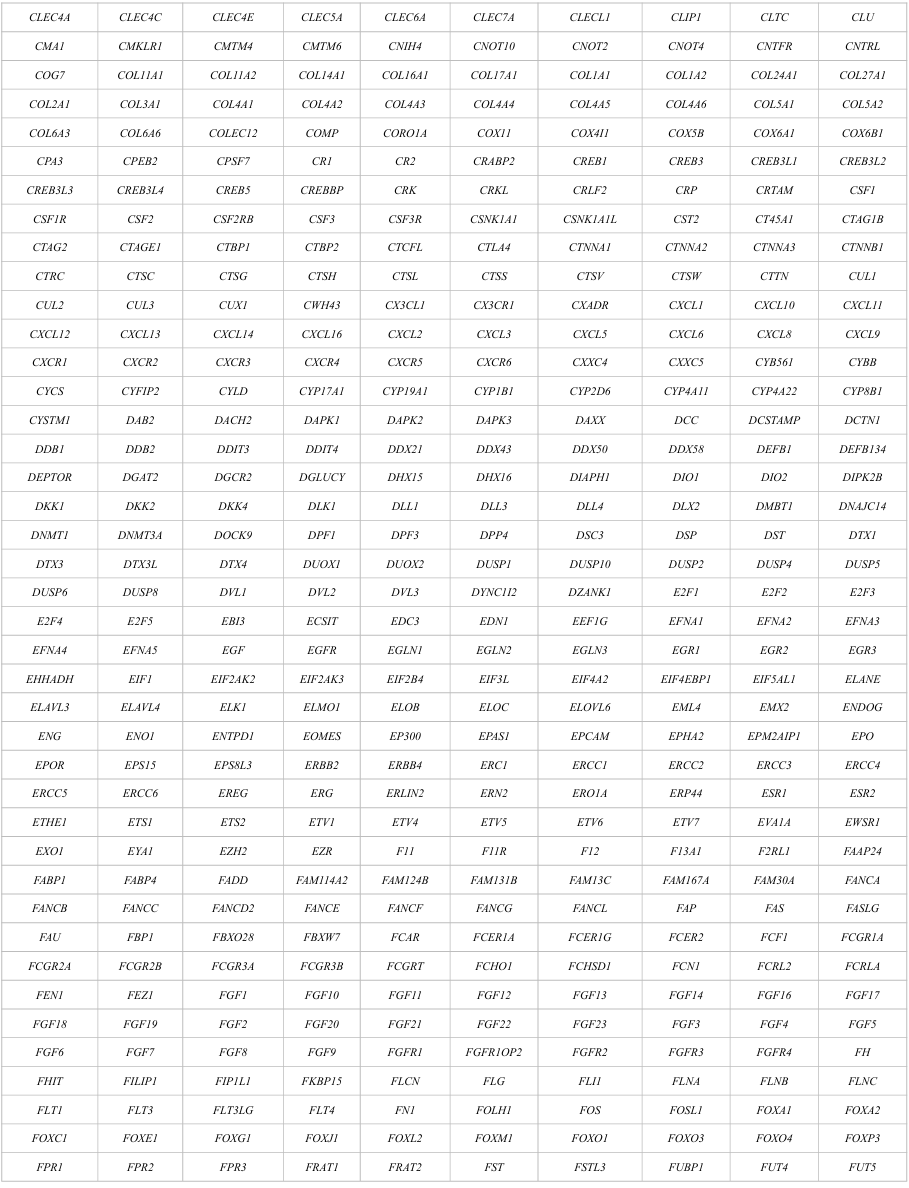

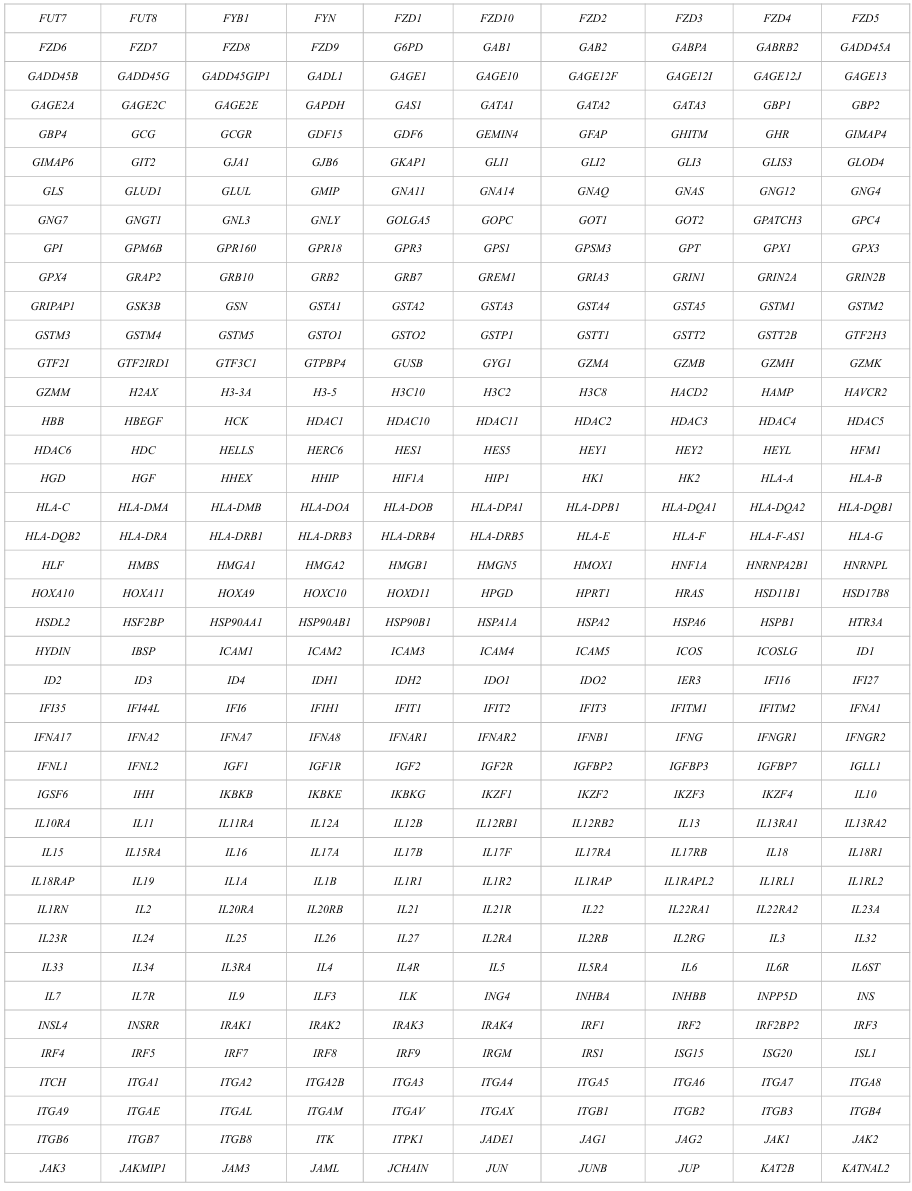

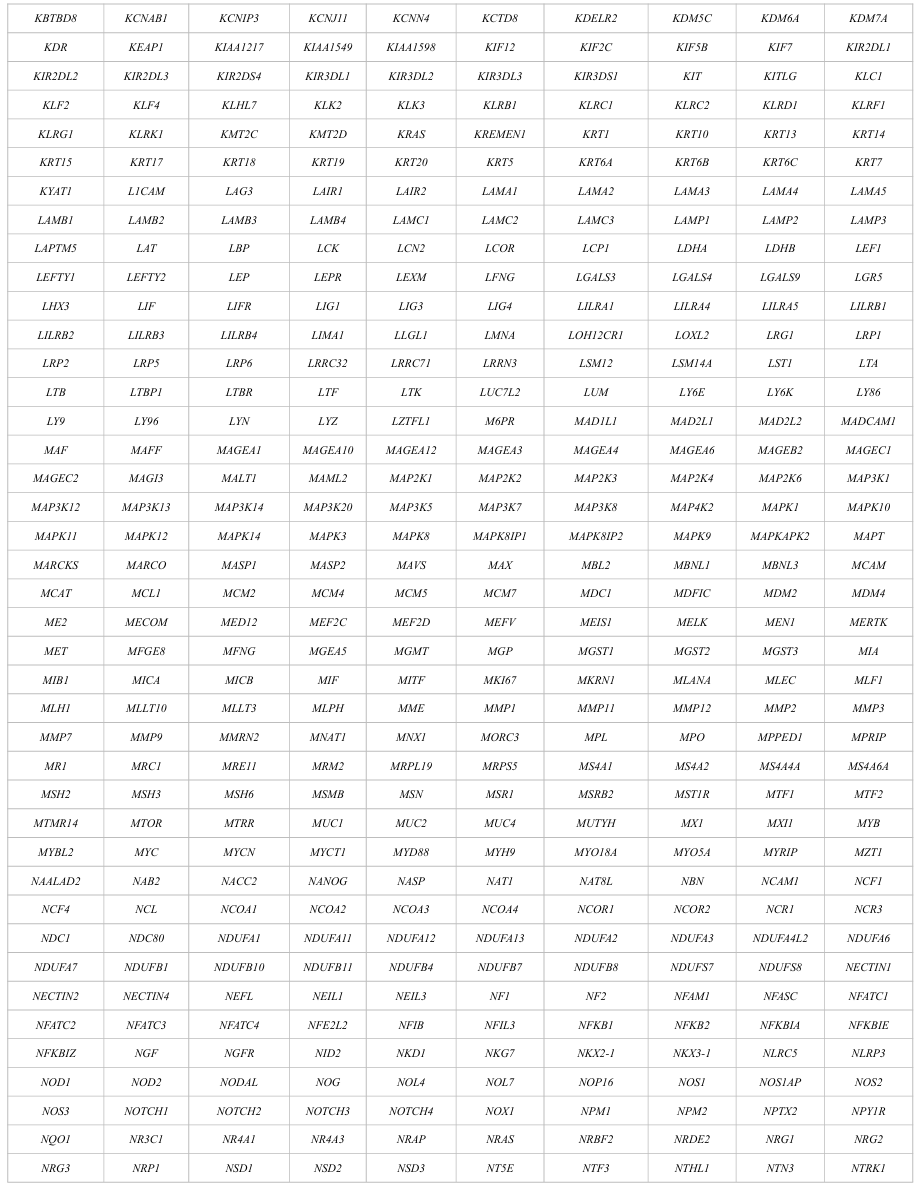

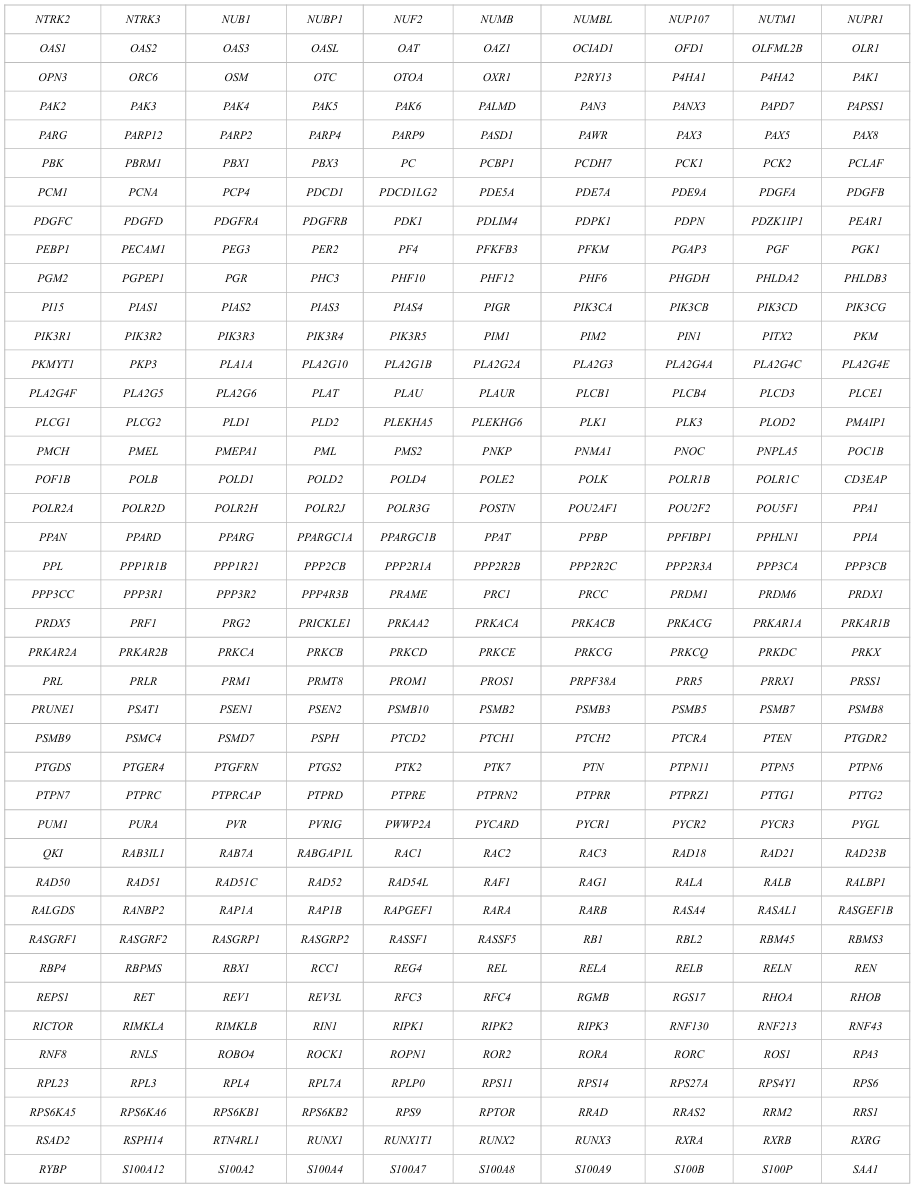

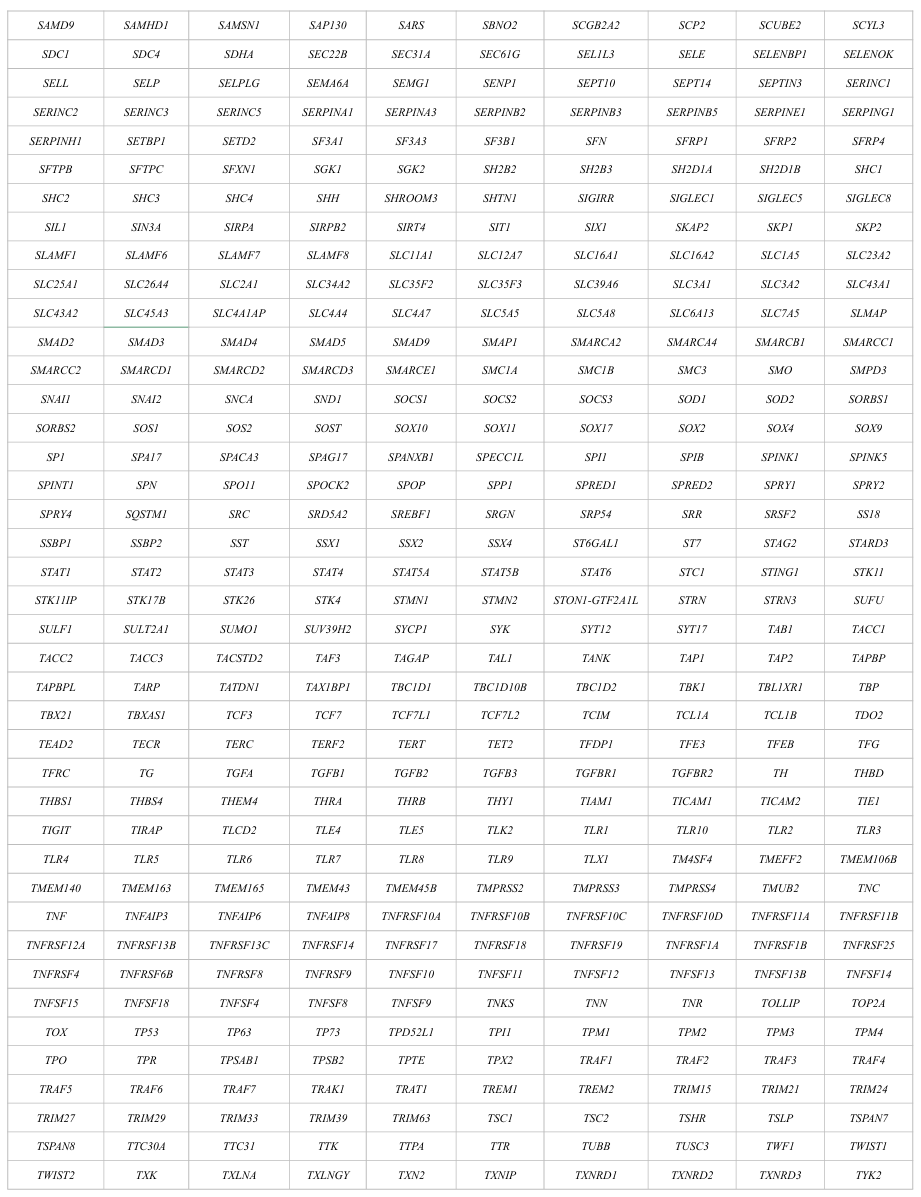

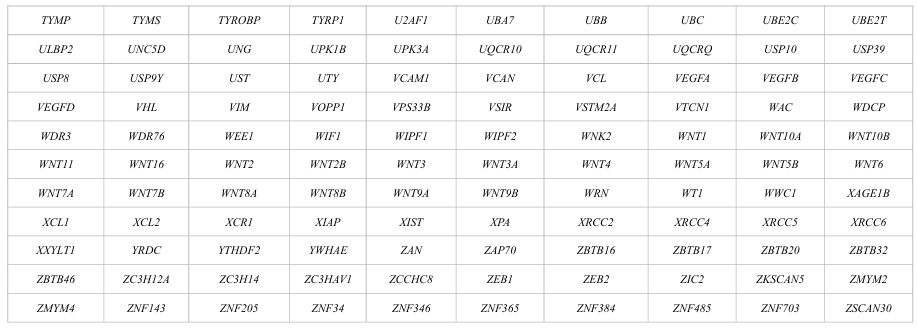

Supplement: Supplementary file 1 [file Table1.docx]
