# Supplementary material for: Case Report: Unclassifiable cerebellar high-grade neuroepithelial tumor with a CCDC6::RET fusion manifesting explosive recurrence
Source: Front Surg. 2026 Apr 8;13:1709333. doi: 10.3389/fsurg.2026.1709333 (PMC13099530; doi:10.3389/fsurg.2026.1709333)
Supplement: Supplementary file 2 [file Table2.docx]

**Supplementary Table S1. Targeted DNA and RNA sequencing results**

**S1A. Assay and sample details**

| **Field** | **Value** |
| --- | --- |
| Specimen | FFPE scroll (paraffin roll) |
| Panel coverage | DNA: 571 genes; RNA: 2660 genes |
| Methods | Next-generation sequencing, hybrid-capture targeted panel |
| Analytes | DNA and RNA extracted from FFPE tissue |
| Quality control | Passed |

**S1B. Clinically relevant alterations**

| **Alteration** | **Details** | **Result** | **Quantification** |
| --- | --- | --- | --- |
| RET fusion | CCDC6::RET (CCDC6 exon 1 to RET exon 12) | Detected | Abundance 89.24% (1120 supporting reads) |
| TP53 | c.524G>A (p.R175H), exon 5 missense | Detected | Variant allele fraction 80.23% |
| CDK4 amplification | Copy number gain | Detected | Copy number reported as 9 |
| AURKA amplification | Copy number gain | Detected | Copy number reported as 9 |

**S1C. Other detected tumor variants**

| **Gene** | **Alteration** | **Variant type** | **Quantification** |
| --- | --- | --- | --- |
| ARID1A | c.1224G>A (p.P408=), exon 2 synonymous | SNV | VAF 45.35% |
| ASXL1 | c.3416C>A (p.T1139K), exon 13 missense | SNV | VAF 50.27% |
| AXIN2 | c.1516G>A (p.V506M), exon 6 missense | SNV | VAF 51.36% |
| CDK8 | c.-15T>A, exon 1 (5' region) | SNV | VAF 7.67% |
| MUTYH | c.850-2A>G, intronic splice-site | SNV | VAF 49.42% |
| PLCG2 | c.3264G>T (p.E1088D), exon 29 missense | SNV | VAF 8.14% |
| TSC1 | c.1460C>G (p.S487C), exon 15 missense | SNV | VAF 84.70% |

**S1D. Biomarkers reported by the assay**

| **Biomarker** | **Result** |
| --- | --- |
| Microsatellite instability (MSI) | MSI-H not detected |
| Tumor mutational burden (TMB) | 5.83 mutations/Mb (low) |
| Gene expression profile (GEP) score | -1.6 |
| Tumor microenvironment (TME) subtype | Immune desert (D) |
| Homologous recombination deficiency (HRD) | Negative |
| Genomic scar score (GSS) | 37.3 (positive cutoff: 45) |
| BRCA1 | No pathogenic or likely pathogenic variants detected |
| BRCA2 | No pathogenic or likely pathogenic variants detected |

*Abbreviations: FFPE, formalin-fixed paraffin-embedded; VAF, variant allele fraction; MSI, microsatellite instability; TMB, tumor mutational burden; GEP, gene expression profile; TME, tumor microenvironment; HRD, homologous recombination deficiency; GSS, genomic scar score.*
